# Cervical intraepithelial neoplasia and cervical cancer in Hunan Province, China, 2020-2023

**DOI:** 10.3389/fonc.2024.1480983

**Published:** 2024-12-04

**Authors:** Xu Zhou, Ting Han, Tingting Guo, Yaqin Liu, Hongyun Li, Wang Yingxia, Yinglan Wu

**Affiliations:** Department of Women Health Care, Hunan Provincial Maternal and Child Health Care Hospital, Hunan, China

**Keywords:** cervical cancer, cervical intraepithelial neoplasia, incidence, pelvic examination, human papillomavirus

## Abstract

**Objective:**

To describe the incidence and distribution of cervical intraepithelial neoplasia (CIN) and cervical cancer (CC) for rural women aged 35-64 in Hunan Province, China, 2020-2023.

**Methods:**

Data were from the Hunan Provincial Cervical Cancer Screening Program in Rural Areas. Most rural women aged 35-64 in Hunan Province attend the program. All women diagnosed with CINs and CCs will be asked to register detailed information, including pelvic examination, diagnosis, age, education level, etc. CINs included low-grade squamous intraepithelial lesions (LSIL) (CIN1), high-grade squamous intraepithelial lesions (HSIL) (CIN2 and 3); CCs included adenocarcinoma *in situ* (AIS), early invasive cervical cancer (EICC) (stage Ia1 and Ia2) and invasive cervical cancer (ICC) (stage Ib and above). The incidence of CIN and CC is the number of cases per 1000 women. Chi-square tests (*χ^2^*) were used to examine if there were significant differences in proportions among different groups.

**Results:**

A total of 4150700 women were included, and 67071 CINs and CCs were identified. The incidence of LSIL, HSIL, AIS, EICC and ICC were 10.63‰(95%CI: 10.53-10.73), 4.98‰(95%CI: 4.91-5.05), 0.06‰(95%CI: 0.06-0.07), 0.23‰(95%CI: 0.22-0.25), and 0.26‰(95%CI: 0.24-0.27), respectively. The proportion of previous pelvic examinations was relatively low in EICC (17.24%) and ICC (17.45%) (*χ^2^ =* 236.57, *P <*0.01), present abnormal examination was relatively high in AIS (51.33%), EICC (49.74%) and ICC (62.45%) (*χ^2^ =* 331.15, *P <*0.01). HPV16 was the most common high-risk type for LSIL (22.01%), HSIL (36.92%), AIS (46.01%), EICC (58.88%), and ICC (64.34%). The proportion of HSIL was relatively high in women aged 35-44 (27.03%), AIS was relatively high in women aged 45-54 (46.39%), EICC (44.24%), and ICC (48.58%) was relatively high in women aged 55-64. The proportion of ICC was relatively high in women with elementary school (38.68%), HSIL (15.10%) and AIS (17.49%) was relatively high in women with senior high school, AIS (1.52%), EICC (0.62%) and ICC (0.75%) was relatively low in women with university and above. (*P <*0.01).

**Conclusion:**

We have described the incidence and distribution of CIN and CC among rural women aged 35-64. These findings were clinically relevant and were useful for clinical counseling and early diagnosis of CC.

## 1 Introduction

Cervical cancer (CC) is the fourth most common cancer among women globally (1). Globally in 2020, there were an estimated 604 127 CC cases and 341 831 deaths, with a corresponding age-standardized incidence of 13.3 cases per 100 000 women-years (95% CI 13.3–13.3) and mortality rate of 7.2 deaths per 100 000 women-years (95% CI 7.2–7.3) (2). CC is a global public health problem, with a particularly high burden in many low-income and middle-income countries (2), and is the most frequent gynecological cancer in developing countries (3). CC is also the leading cause of cancer-related deaths among women in low- and middle-income countries. Each year, China accounts for about 1/5 of the world’s CC incidence and deaths (4, 5). Moreover, the incidence and mortality of CC have shown an upward trend in recent years in China (6, 7). It has been one of the major threats to women’s health (6). Persistent high-risk human papillomavirus (HPV) infection is strongly and consistently associated with high-grade cervical intraepithelial neoplasia (CIN) acquisition and is considered essential for the progression of CC (8). Therefore, the study on CIN and CC is significant and deserves more attention.

In the early stages, CC is often asymptomatic; as CC progresses, nonspecific symptoms such as vaginitis and cervicitis may develop (9). Previous studies showed that persistent genital high-risk human papillomavirus (HPV) infection was the leading cause of CC (9). Okunade reported that about 99.7% of CC cases were caused by persistent genital high-risk human papillomavirus (HPV) infection (10). To reduce the incidence and mortality of CC, HPV screening and HPV vaccines for CC are becoming more and more widely used (11–13).

In 2009, the National Health Commission of the People’s Republic of China began to implement China’s National Cervical Cancer Screening Program in Rural Areas in some selected counties, which provides free screening for rural women aged 15-64 years and to encourage early diagnosis, detection, prevention, and treatment of CC (14, 15). In 2012, Hunan Province began implementing the Hunan Provincial Cervical Cancer Screening Program in Rural Areas (HPCCSPRA) in all counties, providing free CC HPV screening, diagnosis, and treatment for rural women aged 35-64.

Although there have been some studies on CC screening, diagnosis, and treatment (3, 16, 17), to the best of our knowledge, there are fewer studies on the incidence and distribution of CIN and CC based on long-term, large-sample population data, especially in Hunan Province, China.

Hunan Province is located in south-central China and covers a population of about 65 million. In this study, we conducted a systematic study using data from the HPCCSPRA (2020-2023) to describe the incidence and distribution of CIN and CC for rural women aged 35-64. This information may be useful for clinical counseling and early diagnosis of CC.

## 2 Methods

### 2.1 Data sources

Data sources were obtained from the HPCCSPRA (2020-2023). Rural women aged 35-64 years volunteered to attend the HPCCSPRA. From 2020 to 2023, most rural women in Hunan Province attend the HPCCSPRA.

The service for HPCCSPRA is as follows: (1) All women who attend the HPCCSPRA will receive pelvic examinations, microscopic detection of vaginal secretions (wet film) or Gram stain test, and high-risk HPV testing. The main items of pelvic examinations include vaginitis (including bacterial vaginitis, candida vaginitis, trichomonas vaginitis, et al.), cervicitis, uterine fibroid, cervical polyp, condyloma acuminatum, et al. (2) Women with high-risk HPV will receive further cervical cytology. If the result of the cervical cytology is abnormal or suspected, they will receive further colposcopy. Women with HPV 16 or 18 will receive colposcopy directly. According to the International Agency for Research on Cancer, of the 30-40 HPV types that infect the anogenital tract, there are 13 high-risk (16, 18, 31, 33, 35, 39, 45, 51, 52, 56, 58, 59, and 68) HPV types (18). (3) Women with abnormal or suspicious colposcopy will receive histopathological examination. (4) All women diagnosed with CINs and CCs will be asked to register detailed information, including previous pelvic examination, present pelvic examination, diagnosis, age, education level, etc. Detailed information about the data collection has been reported elsewhere (19, 20).

### 2.2 Ethics approval and consent to participate

The Hunan Provincial Health Commission routinely collected that data, and the government developed the “Working Manual for HPCCSPRA” to collect that data. Therefore, there is no additional written informed consent. The Medical Ethics Committee of Hunan Provincial Maternal and Child Health Care Hospital approved the study. (NO: 2024-S041). It is a retrospective study of medical records; all data were fully anonymized before we accessed them. Moreover, we de-identified the patient records before analysis. We confirmed that all operations were following relevant guidelines and regulations.

### 2.3 Data quality control

The Hunan Provincial Health Commission developed a quality control manual for the province. Data were collected and reported by experienced and trained doctors and nurses. To ensure data consistency and accuracy, all collectors must be trained and qualified before starting work. The Hunan Provincial Health Commission asks the technical guidance departments to conduct comprehensive quality control yearly to reduce surveillance data integrity and information error rates.

### 2.4 Definitions

According to the International Federation of Gynecology and Obstetrics and Bethesda System for cervical cytology, CINs were classified as low-grade squamous intraepithelial lesions (LSIL) (including CIN1), high-grade squamous intraepithelial lesions (HSIL) (including CIN2 and 3); CCs were classified as adenocarcinoma *in situ* (AIS), early invasive cervical cancer (EICC) (encompassing: stage Ia1 and Ia2) and invasive cervical cancer (ICC) (encompassing: stage Ib and above) according to the types and stages (21, 22). The incidence of CIN and CC is defined as the number of cases per 1000 women. Since almost all CINs and CCs are associated with HPV, the incidence in this study is similar to the actual incidence.

### 2.5 Statistical analysis

The incidence of CIN and CC with 95% confidence intervals (CI) was calculated by the log-binomial method (23). Chi-square tests (*χ^2^*) were used to examine if there were significant differences in proportion among different groups.

Statistical analyses were performed using SPSS 22.0 (IBM Corp., NY, USA).

## 3 Results

### 3.1 Incidence of CIN and CC in Hunan Province, China, 2020-2023

**Figure 1:**
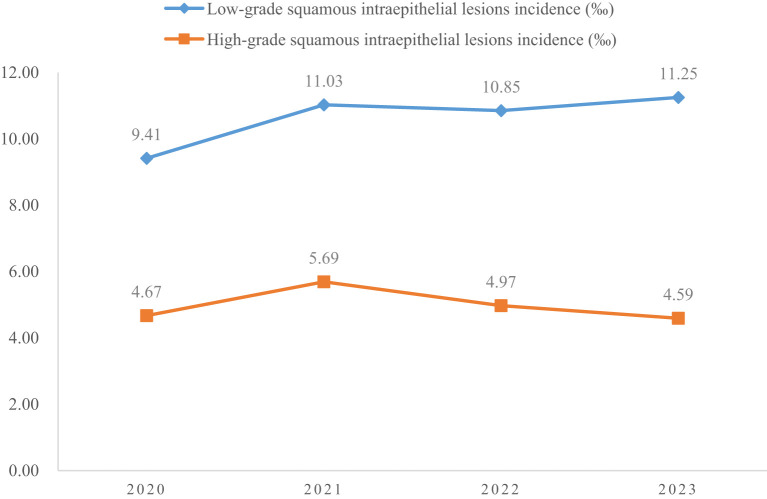
Incidence of cervical intraepithelial neoplasia in Hunan Province, China, 2020-2023.

**Figure 2:**
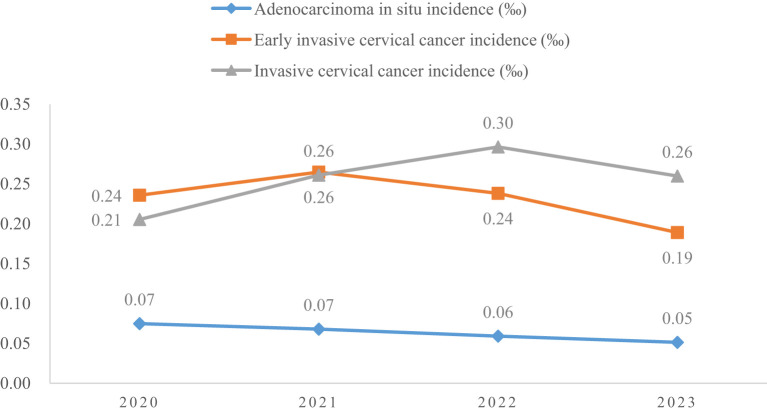
Incidence of cervical cancer in Hunan Province, China, 2020-2023.

A total of 4150700 women were included in this study, and 67071 CINs and CCs were identified. The incidence of LSIL, HSIL, AIS, EICC and ICC were 10.63‰(95%CI: 10.53-10.73), 4.98‰(95%CI: 4.91-5.05), 0.06‰(95%CI: 0.06-0.07), 0.23‰(95%CI: 0.22-0.25), and 0.26‰(95%CI: 0.24-0.27), respectively. The incidence of CIN (including LSIL and HSIL) was 15.61‰(95%CI: 15.49-15.73), and the incidence of CC (including AIS, EICC, and ICC) was 0.55‰ (95%CI: 0.53-0.57). **Table 1** shows the details of the incidence of CIN and CC in Hunan Province, China, 2020-2023. (**Table 1**; **Figures 1**, **2**).

**Table 1:** Incidence of CIN and CC in Hunan Province, China, 2020-2023.

Year	Women	LSIL	Incidence (‰, 95%CI)	HSIL	Incidence (‰, 95%CI)	AIS	Incidence (‰, 95%CI)	EICC	Incidence (‰, 95%CI)	ICC	Incidence (‰, 95%CI)
2020	1056138	9942	9.41 (9.23-9.60)	4936	4.67 (4.54-4.80)	79	0.07 (0.06-0.09)	249	0.24 (0.21-0.27)	217	0.21 (0.18-0.23)
2021	1030455	11363	11.03 (10.82-11.23)	5865	5.69 (5.55-5.84)	70	0.07 (0.05-0.08)	273	0.26 (0.23-0.30)	269	0.26 (0.23-0.29)
2022	1032618	11205	10.85 (10.65-11.05)	5133	4.97 (4.83-5.11)	61	0.06 (0.04-0.07)	246	0.24 (0.21-0.27)	306	0.30 (0.26-0.33)
2023	1031489	11604	11.25 (11.05-11.45)	4737	4.59 (4.46-4.72)	53	0.05 (0.04-0.07)	195	0.19 (0.16-0.22)	268	0.26 (0.23-0.29)
Total	4150700	44114	10.63 (10.53-10.73)	20671	4.98 (4.91-5.05)	263	0.06 (0.06-0.07)	963	0.23 (0.22-0.25)	1060	0.26 (0.24-0.27)

CIN, cervical intraepithelial neoplasia; CC, cervical cancer; LSIL, low-grade squamous intraepithelial lesions; HSIL, high-grade squamous intraepithelial lesions; AIS, adenocarcinoma *in situ*; EICC, early invasive cervical cancer; ICC, invasive cervical cancer.

### 3.2 Pelvic examinations of CIN and CC

**Figure 3:**
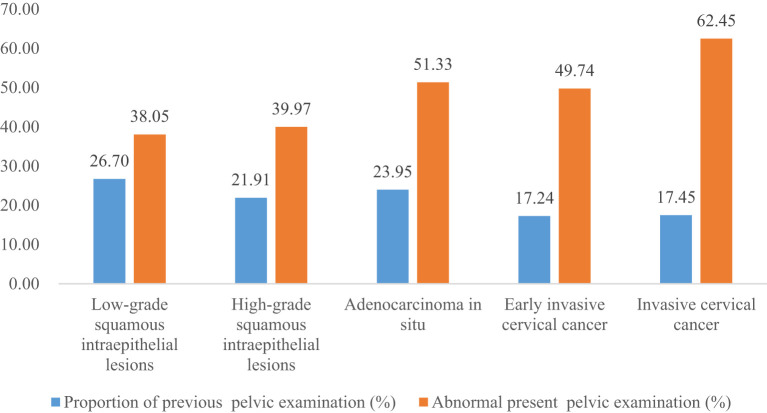
Pelvic examination of cervical intraepithelial neoplasia and cervical cancer.

**Figure 4:**
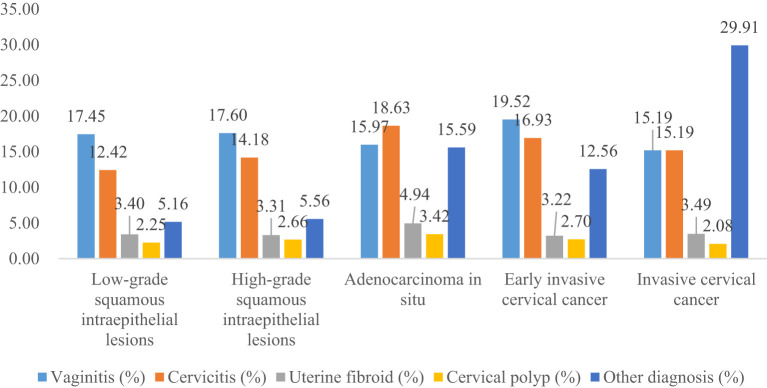
Present clinical diagnosis of cervical intraepithelial neoplasia and cervical cancer.

The proportion of previous pelvic examinations in LSIL, HSIL, AIS, EICC, and ICC was 26.70%, 21.91%, 23.95%, 17.24%, and 17.45%, respectively, and was relatively low in EICC and ICC (*χ^2^ =* 236.57, *P <*0.01). In the present pelvic examination, the proportion of abnormal examination results in LSIL, HSIL, AIS, EICC, and ICC was 38.05%, 39.97%, 51.33%, 49.74%, and 62.45%, respectively, and was relatively high in AIS, EICC and ICC (*χ^2^ =* 331.15, *P <*0.01). In the present clinical diagnosis, the proportion of vaginitis in LSIL, HSIL, AIS, EICC, and ICC was 17.45%, 17.60%, 15.97%, 19.52%, and 15.19%, respectively, without significant differences (*χ^2^ =* 7.30, *P* =0.12); The proportion of cervicitis in LSIL, HSIL, AIS, EICC, and ICC was 12.42%, 14.18%, 18.63%, 16.93%, and 15.19%, respectively, and was relatively high in AIS, EICC, and ICC (*χ^2^ =* 62.66, *P <*0.01); The proportion of uterine fibroid in LSIL, HSIL, AIS, EICC, and ICC was 3.40%, 3.31%, 4.94%, 3.22%, and 3.49%, respectively, without significant differences (*χ^2^ =* 2.46, *P* =0.65); The proportion of cervical polyps in LSIL, HSIL, AIS, EICC, and ICC was 2.25%, 2.66%, 3.42%, 2.70%, and 2.08%, respectively, and was relatively high in AIS (*χ^2^ =* 12.23, *P* =0.02). **Table 2** shows the details of the pelvic examination of CIN and CC. (**Table 2**; **Figures 3**, **4**).

**Table 2:** Pelvic examination and diagnosis of CIN and CC.

Variables	LSIL	Proportion (%)	HSIL	Proportion (%)	AIS	Proportion (%)	EICC	Proportion (%)	ICC	Proportion (%)	*χ^2^*	*P*
Total cases	44114	65.77	20671	30.82	263	0.39	963	1.44	1060	1.58		
Previous pelvic examination												
Yes	11777	26.70	4529	21.91	63	23.95	166	17.24	185	17.45	236.57	<0.01
Within three years	5075	11.50	1811	8.76	18	6.84	77	8.00	59	5.57		
Over three years	6702	15.19	2718	13.15	45	17.11	89	9.24	126	11.89		
No	32337	73.30	16142	78.09	200	76.05	797	82.76	875	82.55		
Present pelvic examination												
Normal	27330	61.95	12409	60.03	128	48.67	484	50.26	398	37.55	331.15	<0.01
Abnormal	16784	38.05	8262	39.97	135	51.33	479	49.74	662	62.45		
Present clinical diagnosis												
Vaginitis	7698	17.45	3639	17.60	42	15.97	188	19.52	161	15.19	7.30	0.12
Bacterial vaginitis	2644	5.99	1104	5.34	15	5.70	60	6.23	46	4.34		
Candida vaginitis	881	2.00	441	2.13	7	2.66	13	1.35	13	1.23		
Trichomonas vaginitis	431	0.98	201	0.97	3	1.14	9	0.93	4	0.38		
Other vaginitis	3860	8.75	1937	9.37	17	6.46	108	11.21	99	9.34		
Cervicitis	5480	12.42	2932	14.18	49	18.63	163	16.93	161	15.19	62.66	<0.01
Mucopurulent cervicitis	920	2.09	711	3.44	19	7.22	69	7.17	50	4.72		
Other cervicitis	4562	10.34	2222	10.75	30	11.41	94	9.76	111	10.47		
Uterine fibroid	1500	3.40	684	3.31	13	4.94	31	3.22	37	3.49	2.46	0.65
Cervical polyp	991	2.25	549	2.66	9	3.42	26	2.70	22	2.08	12.23	0.02
Other diagnosis	2277	5.16	1149	5.56	41	15.59	121	12.56	317	29.91		

CIN, cervical intraepithelial neoplasia; CC, cervical cancer; LSIL, low-grade squamous intraepithelial lesions; HSIL, high-grade squamous intraepithelial lesions; AIS, adenocarcinoma *in situ*; EICC, early invasive cervical cancer; ICC, invasive cervical cancer.

### 3.3 High-risk HPV types in CIN and CC

**Figure 5:**
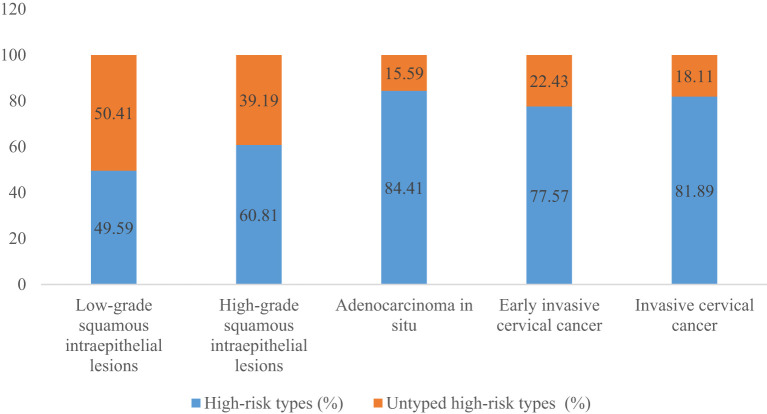
High-risk HPV types in cervical intraepithelial neoplasia and cervical cancer.

**Figure 6:**
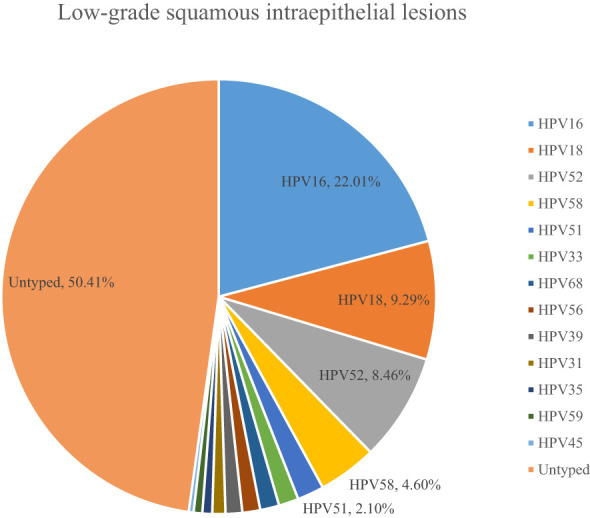
High-risk HPV types in low-grade squamous intraepithelial lesions.

**Figure 7:**
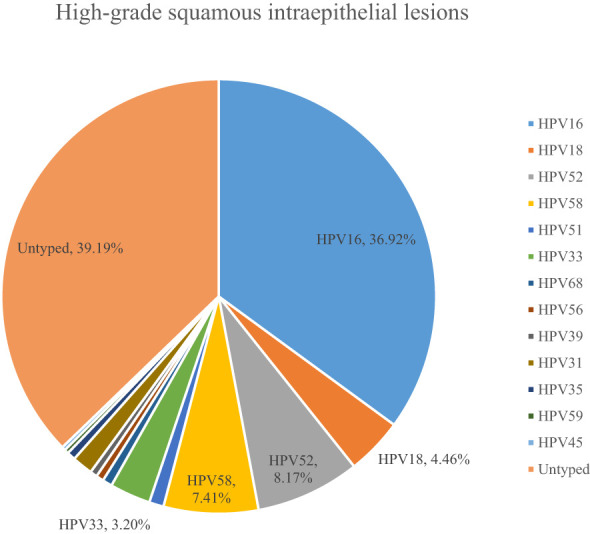
High-risk HPV types in high-grade squamous intraepithelial lesions.

**Figure 8:**
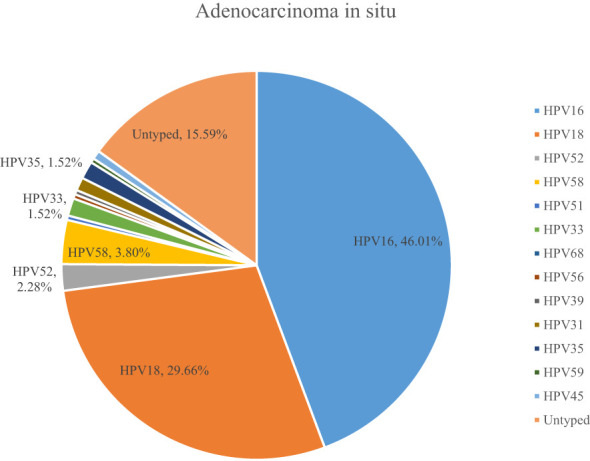
High-risk HPV types in adenocarcinoma in situ.

**Figure 9:**
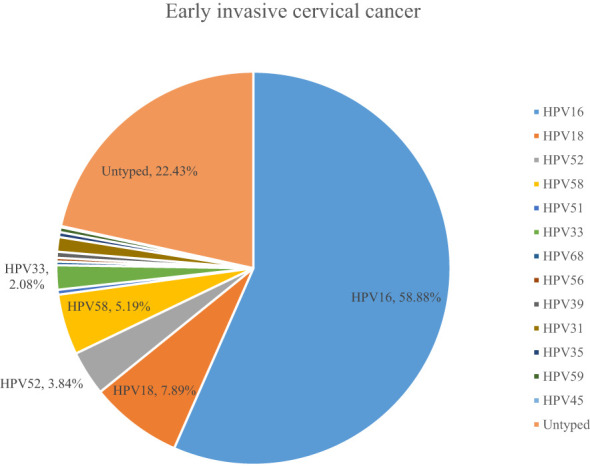
High-risk HPV types in early invasive cervical cancer.

**Figure 10:**
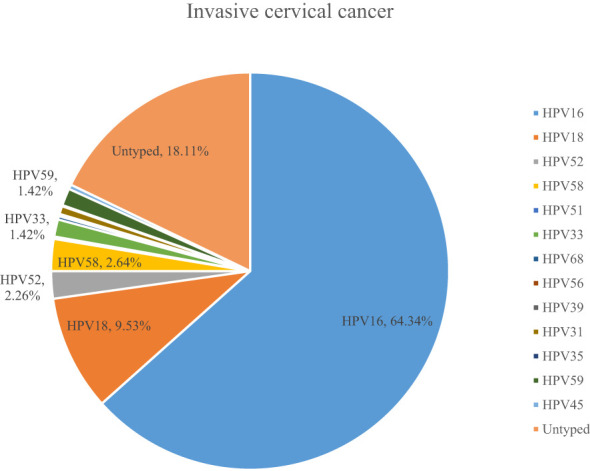
High-risk HPV types in invasive cervical cancer.

A total of 54.10% (36286/67071) of CINs and CCs identified a specific high-risk HPV type. The proportion of untyped high-risk types in LSIL, HSIL, AIS, EICC, and ICC was 50.41%, 39.19%, 15.59%, 22.43%, and 18.11%, respectively, and was relatively high in LSIL and HSIL (*χ^2^ =* 1376.50, *P <*0.01). HPV16 was the most common high-risk type for LSIL (22.01%), HSIL (36.92%), AIS (46.01%), EICC (58.88%), and ICC (64.34%). There were significant differences in the proportions (*χ^2^ =* 2799.41, *P <*0.01). The proportion of HPV16 was relatively high for AIS, EICC, and ICC. HPV18 was the second most common high-risk type for LSIL (9.29%), AIS (29.66%), EICC (7.89%), and ICC (9.53%), and was the fourth most common high-risk type for HSIL (4.46%). There were also significant differences in the proportions (*χ^2^ =* 631.12, *P <*0.01). The proportion of HPV18 was relatively high for AIS. HPV52 (8.17%) and HPV58 (7.41%) were the second and third most common high-risk types for HSIL, respectively, and the proportions were significantly higher than HPV18 (4.46%) (*χ^2^ =* 255.55, *P <*0.01). **Table 3** shows the details of high-risk HPV types in CINs and CCs. (**Table 3**; **Figures 5**–**10**).

**Table 3:** High-risk HPV types in CIN and CC.

HPV types	LSIL	Proportion (%)	HSIL	Proportion (%)	AIS	Proportion (%)	EICC	Proportion (%)	ICC	Proportion (%)
Total cases	44114		20671		263		963		1060	
**High-risk types**	21878	49.59	12571	60.81	222	84.41	747	77.57	868	81.89
HPV16	9708	22.01	7631	36.92	121	46.01	567	58.88	682	64.34
HPV18	4096	9.29	921	4.46	78	29.66	76	7.89	101	9.53
HPV52	3734	8.46	1689	8.17	6	2.28	37	3.84	24	2.26
HPV58	2028	4.60	1532	7.41	10	3.80	50	5.19	28	2.64
HPV51	927	2.10	235	1.14	1	0.38	4	0.42	2	0.19
HPV33	690	1.56	661	3.20	4	1.52	20	2.08	15	1.42
HPV68	657	1.49	157	0.76	0	0.00	3	0.31	3	0.28
HPV56	616	1.40	121	0.59	1	0.38	3	0.31	2	0.19
HPV39	578	1.31	115	0.56	1	0.38	5	0.52	0	0.00
HPV31	455	1.03	334	1.62	3	1.14	12	1.25	7	0.66
HPV35	338	0.77	136	0.66	4	1.52	4	0.42	1	0.09
HPV59	298	0.68	68	0.33	1	0.38	4	0.42	15	1.42
HPV45	169	0.38	59	0.29	2	0.76	1	0.10	4	0.38
**Untyped high-risk types**	22236	50.41	8100	39.19	41	15.59	216	22.43	192	18.11
High-risk types except 16 or 18	16487	37.37	6001	29.03	25	9.51	132	13.71	106	10.00
Other untyped high-risk types	5749	13.03	2099	10.15	16	6.08	84	8.72	86	8.11

CIN, cervical intraepithelial neoplasia; CC, cervical cancer; LSIL, low-grade squamous intraepithelial lesions; HSIL, high-grade squamous intraepithelial lesions; AIS, adenocarcinoma *in situ*; EICC, early invasive cervical cancer; ICC, invasive cervical cancer. Bold values means including any of the following subtypes.

### 3.4 Distribution of CIN and CC by epidemiological characteristics

Women aged 35-44, 45-54, and 55-64 years accounted for 22.61%, 41.20%, and 36.19% of LSIL, respectively. Compared to LSIL, the proportion of HSIL was relatively high in women aged 35-44 years (27.03%), the proportion of AIS was relatively high in women aged 45-54 years (46.39%), the proportion of EICC (44.24%) and ICC (48.58%) was relatively high in women aged 55-64 years. (*P <*0.01).

**Figure 11:**
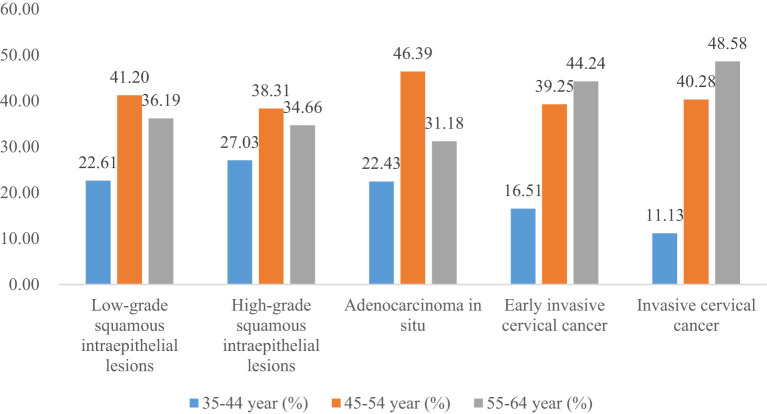
Distribution of cervical intraepithelial neoplasia and cervical cancer by age.

**Figure 12:**
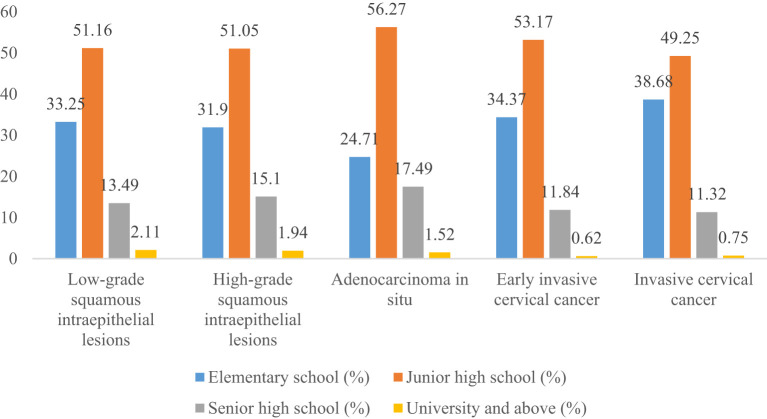
Distribution of cervical intraepithelial neoplasia and cervical cancer by education level.

Women with elementary school, junior high school, senior high school, and university and above accounted for 33.25%, 51.16%, 13.49%, and 2.11% of all LSIL, respectively. Compared to LSIL, the proportion of ICC was relatively high in women with elementary school (38.68%), the proportion of HSIL (15.10%) and AIS (17.49%) was relatively high in women with senior high school, the proportion of AIS (1.52%), EICC (0.62%) and ICC (0.75%) was relatively low in women with university and above. (*P <*0.01). (**Table 4**; **Figures 11**, **12**).

**Table 4:** Distribution of CIN and CC by epidemiological characteristics.

Variables	LSIL	Proportion (%)	HSIL	Proportion (%)	AIS	Proportion (%)	EICC	Proportion (%)	ICC	Proportion (%)	*χ^2^*	*P*
Total cases	44114		20671		263		963		1060			
Age (year)												
35-44	9976	22.61	5587	27.03	59	22.43	159	16.51	118	11.13	275.69	<0.01
45-54	18174	41.20	7919	38.31	122	46.39	378	39.25	427	40.28	53.31	<0.01
>55-64	15964	36.19	7165	34.66	82	31.18	426	44.24	515	48.58	120.60	<0.01
Education level												
Elementary school	14668	33.25	6595	31.90	65	24.71	331	34.37	410	38.68	36.69	<0.01
Junior high school	22568	51.16	10553	51.05	148	56.27	512	53.17	522	49.25	5.95	0.20
Senior high school	5949	13.49	3121	15.10	46	17.49	114	11.84	120	11.32	43.09	<0.01
University and above	929	2.11	402	1.94	4	1.52	6	0.62	8	0.75	20.70	<0.01

CIN, cervical intraepithelial neoplasia; CC, cervical cancer; LSIL, low-grade squamous intraepithelial lesions; HSIL, high-grade squamous intraepithelial lesions; AIS, adenocarcinoma *in situ*; EICC, early invasive cervical cancer; ICC, invasive cervical cancer.

## 4 Discussion

Overall, we have described the incidence and distribution of CIN and CC among rural women aged 35-64. To the best of our knowledge, previous studies rarely address this issue. The study was clinically relevant, and our findings may be useful for clinical counseling and early diagnosis of CC.

First, we have reported the incidence of CIN and CC for rural women aged 35-64. To the best of our knowledge, few studies have reported the incidence of CIN and CC for rural women aged 35-64 years based on a large population, including in China. Some studies have reported the overall incidence of CC, or the incidence of CIN, for women aged 35-64. For example, Gu et al. reported that the CC incidence was 0.28‰ for the total population aged 35-64 years in China (2000-2015) (24), and the CC incidence for women aged 35-64 years was about 0.56‰ (half of the total). It is consistent with the CC incidence reported in this study. Zhao et al. reported that the CC incidence was 26.54/100000, and the HSIL incidence was 223.89/100000 for women aged 35-64 years in rural areas in China (2018) (19); Shen et al. reported that the incidence of HSIL and CC was 395.6/100000 for women aged 35-64 years in Beijing City, China (2014-2015) (25); Lu et al. reported that the incidence of LSIL, HSIL, and CC was 1.89%, 0.60% and 0.03%, respectively, for women in minority area, Guangxi Province, China (2012-2019) (26); Shahmoradi et al. reported that the CC incidence among US women aged 35-64 years was from 14.38/100000 to 25.51/100000 (2001-2019) (27); Cleveland et al. reported that AIS incidence for women aged 40-64 years was about 2.4/100000 to 3.4/100000 (US, 2008-2015) (28). There are significant differences between these studies and the present study. On the one hand, it may be associated with differences in economic and medical conditions in different areas, which may affect CC screening and diagnosis rates (29). On the other hand, some previous studies were limited in study data, such as the study populations were from several hospitals, from small areas, or over a shorter period. To the best of our knowledge, no study has reported the incidence of CIN and CC in Hunan Province based on province-wide data. In addition, we have reported the incidence of CIN and CC by stages. It is of great significance for public health.

Second, among LSIL, HSIL, AIS, EICC, and ICC, there were significant differences in the proportion of previous pelvic examination and abnormal present pelvic examination, with a relatively low proportion of previous pelvic examination in EICC and ICC and a relatively high proportion of abnormal present pelvic examination in AIS, EICC, and ICC. It suggests that the more severe the CC, the more common the associated abnormal pelvic examinations are, but the lower the proportion of pelvic examinations. Moreover, it also suggests that women suffering from CC may be associated with a lack of pelvic examination. Previous studies support these findings (30). In this study, among LSIL, HSIL, AIS, EICC, and ICC, there was no significant difference in the proportion of vaginitis and uterine fibroid. At the same time, there were significant differences in the proportion of cervicitis and cervical polyp. The proportion of cervicitis was relatively high in AIS, EICC, and ICC, and the proportion of cervical polyps was relatively high in AIS. Overall, fewer studies address the above issues.

Although details of the negative population were not available in this study, if we regard LSIL as the reference, some previous studies were consistent with this study. For example, Long et al. reported that severe cervical inflammation was positively related to HSIL (31). Zhang et al. reported that cervical inflammation was a risk factor for cervical lesions (32). Zhao et al. reported that concurrent reproductive tract infections (such as cervicitis and cervical erosion) were risk factors for CC (33). Levy et al. reported an increased incidence of atypical squamous cells of undetermined significance, and atypical glandular cells not otherwise specified pap diagnoses were found in women with benign polyps on biopsy (34). Kucukyıldız et al. reported that endocervical polyps may be more common in patients with high-risk HPV infections (35). However, some previous studies were inconsistent with this study. For example, Brusselaers et al. found a causal link between vaginal dysbiosis and CC along the oncogenic human papillomavirus acquisition, persistence, and cervicovaginal dysplasia development pathway (36). Tao et al. reported that risk factors for HSIL included both Trichomonas vaginalis infection and cervical inflammation (37). The inconsistent results described above may be related to various factors (30, 38), such as different economic and medical conditions. Our findings suggest that there may be a correlation, perhaps a causal relationship, between CIN and CC and a broad range of abnormal pelvic examinations. It deserves in-depth research. Our findings may be useful for future in-depth studies.

Third, previous studies showed that almost all CCs are associated with HPV infection (10). In this study, we have presented the 13 high-risk HPV types for CIN and CC in detail, which have rarely been addressed in previous studies. HPV16 and HPV 18 were the most common high-risk types for all stages except for HSIL, and the more severe the CC, the higher the proportion of HPV16. It is consistent with previous studies (39, 40). However, there were significant differences in HPV types and proportions between this study and previous studies. For example, Muñoz et al. reported that HPV45 (4.4%) and HPV31 (3.4%) were common high-risk types (11 studies in nine countries, from 1985 to 1997) (40). Yang et al. reported that the most common high-risk HPV types were HPV52 (22.02%), HPV58 (12.79%) and HPV16 (12.60%) in LSIL; HPV16 (28.31%), HPV52 (20.63%), and HPV58 (17.73%) in HSIL; HPV16 (37.17%), HPV52 (13.77%), and HPV18 (8.39%) in EICC and ICC (Guangzhou City, China, 2015-2021) (41). Zhang et al. reported that the most common high-risk HPV types were HPV52 (20.31%), HPV16 (16.81%), HPV58 (14.44%), HPV18 (6.44%), and HPV53 (5.76%) in CIN1 (LSIL); HPV16 (45.69%), HPV58 (15.50%), HPV52 (11.74%), HPV33 (9.35%), and HPV31 (4.34%) in CIN2/3 (HSIL) (China, 2000-2019) (42). Cleveland et al. reported that HPV16 was the most frequently detected high-risk type in both AIS (57%) and CIN3 (58%), whereas HPV18 was the second most common high-risk type in AIS (38%) and less common in CIN3 (5%) (US, 2008-2015) (28). It is inconsistent with this study. These differences may be related partly to the fact that detailed HPV types were not reported in many cases, especially in LSIL and HSIL. It may lead to difficulties in comparing this study with other studies. It is a shortcoming in this study. However, due to the large sample size and comprehensive presentation of various HPV types in this study, this remains an important reference, for example, for vaccine research (43).

Fourth, compared to LSIL, the proportion of EICC and ICC was relatively high in women aged 55-64; the proportion of AIS, EICC, and ICC was relatively low in women with university and above. Details of the negative population were unavailable in this study. If we regard LSIL as the reference, we would get the following findings: the proportion of severe CC was relatively high in the older age groups and relatively low in women with high education levels. It is consistent with previous studies (26, 32, 37, 44, 45). The proportion of severe CC was relatively high in the older age groups, possibly associated with several factors. On the one hand, in the early stages, CC is often asymptomatic, resulting in most women not being screened or examined (9, 46). When severe CC appears, it often develops over a long time. On the other hand, women’s economic conditions, medical conditions, and health knowledge increase with age, increasing CC screening rates (4, 29, 47, 48). Compared to older women, younger women may lack systematic screening and pelvic examination. The proportion of severe CC was relatively low in highly educated people, which may be mainly associated with better health knowledge and economic conditions. Health knowledge is one of the main factors in CC screening; a higher level of education and confidence that any potential symptom would be identified were associated with increased awareness (47–50). Education level is associated with economic conditions, which may have an important impact on CC screening, diagnosis, and treatment (29, 51). Women with high education levels may access earlier screening and diagnosis of CC, reducing the proportion of severe CC.

Some things could be improved in this study. First, due to data limitations, some demographic characteristics, such as economic conditions and ethnic groups, were not included in this study. In addition, there is no detailed information on the medical cost of CIN and CC. Second, detailed HPV types were not reported in many patients, especially in LSIL and HSIL, which may lead to difficulties in comparing this study with other studies. Third, this study focused on the pelvic examination and epidemiology of patients. Due to the lack of detailed information on the negative population, we could not conduct some analyses, such as calculating the incidence of CIN and CC by age and education level and analyzing risk factors for CIN and CC. Fourth, all the patients in this study were women with high-risk HPV. However, although almost all CINs and CCs are associated with high-risk HPV, there are still some that may not be associated with high-risk HPV. Therefore, the incidence of CIN and CC may be slightly underestimated.

Despite some limitations, this study is still of great significance. On the one hand, this study involved many people and covered a wide area, which was well representative. On the other hand, the research limitations pointed out above also provided directions for further in-depth research, and our findings may be useful for future in-depth studies.

## 5 Conclusion

We have described the incidence and distribution of CIN and CC among rural women aged 35-64. These findings were clinically relevant and were useful for clinical counseling and early diagnosis of CC.

## Data Availability

The original contributions presented in the study are included in the article/supplementary material. Further inquiries can be directed to the corresponding author/s.
